# *nab*-Paclitaxel dose and schedule in breast cancer

**DOI:** 10.1186/s13058-015-0587-y

**Published:** 2015-06-12

**Authors:** Miguel Martín

**Affiliations:** Medical Oncology Service, Hospital General Universitario Gregorio Marañón, Instituto de Investigación Sanitaria Gregorio Marañón, Universidad Complutense, Dr Esquerdo 46, Madrid, 28007 Spain

## Abstract

*nab*-Paclitaxel is approved for the treatment of metastatic breast cancer on an every-3-week schedule based on positive findings from a pivotal phase III trial in which *nab*-paclitaxel 260 mg/m^2^ every 3 weeks was superior to solvent-based paclitaxel 175 mg/m^2^ every 3 weeks for the primary endpoint of overall response rate (33 % vs 19 %; *P* = 0.001). Subsequently, a number of trials have examined different schedules, doses, and combinations in efforts to optimize *nab*-paclitaxel-based therapy for metastatic and early-stage breast cancer. The goal of this review is to evaluate the clinical experiences to date with *nab-*paclitaxel as a single agent or in combination with targeted agents in different treatment settings - with a focus on the feasibility of administration, adverse event profile, and standard efficacy endpoints, such as overall survival, progression-free survival, overall response rate, and pathologic complete response rate. In general, weekly dosing during the first 3 of 4 weeks appears to achieve the best clinical benefit in both the metastatic and early-stage settings. Furthermore, the data suggest that high doses of *nab*-paclitaxel, such as 150 mg/m^2^ during first 3 of 4 weeks or 260 mg/m^2^ every 2 weeks, may be more feasible and appropriate for treatment of early-stage disease compared with metastatic disease. Intense regimens of *nab*-paclitaxel may not be the best treatment approach for unselected patients with metastatic breast cancer, but may suit a subset of patients for whom immediate disease control is required. The growing number of *nab*-paclitaxel trials in breast cancer will lead to greater refinements in tailoring therapy to patients based on their individual disease and patient characteristics.

## Introduction

Taxanes are among the most widely used chemotherapy agents in the treatment of early-stage and metastatic breast cancer (MBC) [1–4]. Taxanes stabilize microtubules, leading to cell cycle arrest and, ultimately, cell death [5–7]. Guidelines by the National Comprehensive Cancer Network currently list the taxanes solvent-based paclitaxel (sb-paclitaxel, Taxol; Bristol-Myers Squibb Co., Princeton, NJ, USA), docetaxel (Taxotere; sanofi-aventis US LLC, Bridgewater, NJ, USA), and *nab*-paclitaxel (Abraxane; Celgene Corporation, Summit, NJ, USA) as agents recommended for the treatment of recurrent and MBC [1].

sb-Paclitaxel and docetaxel have demonstrated clinical benefit in the treatment of breast cancer (BC); however, their chemical formulations have presented several limitations. Both sb-paclitaxel and docetaxel require the use of solvents to enhance their solubility (sb-paclitaxel is formulated in the castor oil derivative Cremophor EL (recently renamed Kolliphor EL; BASF Corporation, Florham Park, NJ, USA) and dehydrated ethanol, while docetaxel is formulated using the solvent polysorbate 80), and these solvents have been associated with hypersensitivity and other toxicities, including prolonged peripheral neuropathy [8–10]. To ameliorate the risk of hypersensitivity reactions from solvents, patients are routinely pretreated with corticosteroids before receiving either drug [3, 4]. These solvent vehicles also impair drug delivery to the tumor, limiting their clinical effectiveness [10]. Further, sb-paclitaxel displays more-than-dose-proportional increases in systemic drug exposure over a narrow dose range [11–14]. This nonlinear dose-responsiveness pattern is most likely explained by the entrapment of paclitaxel in solvent micelles [14, 15]. In patients with MBC, no significant dose–response relationship was observed with increasing doses of sb-paclitaxel administered every 3 weeks (q3w; response rates of 23 %, 26 %, and 21 % for 175, 210, or 250 mg/m^2^ doses, respectively) [16]. Although time to progression did significantly increase with increasing dose (*P* = 0.045), this effect was not apparent in a multivariate analysis that included dose and covariates such as estrogen receptor status, line of therapy, number of metastatic sites, performance status, and prior treatment. There were no significant differences in overall survival (OS) among the doses, and the increasing dose of sb-paclitaxel resulted in a higher incidence of toxicities, including neutropenia, neuropathy, and alopecia.

*nab*-Paclitaxel, an albumin-bound form of paclitaxel, is solvent free and was designed to improve the therapeutic index of paclitaxel (that is, to increase antitumor activity and reduce toxicities such as hypersensitivity reactions) [2, 17]. Compared with sb-paclitaxel, *nab*-paclitaxel has demonstrated enhanced transport across endothelial cell monolayers and greater tumor delivery of paclitaxel in preclinical models [17]. A phase I dose escalation study of patients with solid tumors determined that the maximum tolerated dose of *nab*-paclitaxel administered q3w was 300 mg/m^2^, and treatment at this dose resulted in two partial responses in patients with BC and prior exposure to sb-paclitaxel [18]. Dose-limiting toxicities included sensory neuropathy, stomatitis, and superficial keratopathy. In a phase II trial of patients with MBC, *nab*-paclitaxel at the maximum tolerated dose q3w resulted in a 48 % overall response rate (ORR) for all patients and a 64 % ORR for chemotherapy-naïve patients [19]. In a pivotal phase III trial of patients with MBC, *nab*-paclitaxel at a slightly lower dose of 260 mg/m^2^ demonstrated superior antitumor activity compared with sb-paclitaxel 175 mg/m^2^ (both administered q3w) [20]. In 2005, *nab*-paclitaxel was approved for the treatment of MBC after failure of combination chemotherapy for metastatic disease or relapse within 6 months of adjuvant chemotherapy [2]. Prior therapy should have included an anthracycline unless clinically contraindicated. The recommended starting dose of *nab*-paclitaxel for the treatment of MBC is 260 mg/m^2^ administered intravenously over 30 min q3w.

Despite a higher drug cost than other taxanes, *nab*-paclitaxel appeared to be cost-effective in health economic studies. In fact, a meta-analysis of clinical and safety data from randomized trials in MBC found that both the number of grade 3/4 toxicities and resulting management costs were lower for *nab*-paclitaxel relative to docetaxel and sb-paclitaxel [21]. The cost-effectiveness of *nab*-paclitaxel in MBC has also been demonstrated in model-based and retrospective analyses [22, 23].

Weekly sb-paclitaxel administration appears to be the optimal schedule for treatment of MBC. Weekly sb-paclitaxel resulted in superior efficacy compared with treatment q3w in patients with MBC (ORR: 42 % vs 29 %, *P* = 0.0004; time to progression: 9 vs 5 months, *P* < 0.0001; and OS: 24 vs 12 months, *P* = 0.0092 for weekly vs q3w, respectively) [24]. This schedule was also superior to treatment q3w in the adjuvant setting after standard administration of doxorubicin plus cyclophosphamide (AC) [25].

Initial evidence suggested that a weekly *nab*-paclitaxel regimen could also be feasible for patients with MBC. A phase I trial of *nab*-paclitaxel administered weekly for 3 consecutive weeks followed by 1 week of rest (that is, during the first 3 of 4 weeks (qw 3/4)) demonstrated the feasibility of this schedule in patients with advanced solid tumors, and established the maximum tolerated doses for this *nab*-paclitaxel regimen to be 100 mg/m^2^ for heavily pretreated patients and 150 mg/m^2^ for lightly pretreated patients (*n* = 39, nine patients with BC) [26]. Dose-limiting toxicities included grade 4 neutropenia and grade 3 peripheral neuropathy, and a partial response was noted in one patient with BC. The positive results observed with weekly sb-paclitaxel and promising results from the phase I trial of *nab*-paclitaxel administered qw 3/4 has led many to question whether weekly dosing might be preferable to a schedule q3w for *nab*-paclitaxel.

Here we review the experience to date with *nab*-paclitaxel-based therapy for the treatment of BC, with the goal of understanding the optimal dose/schedule of *nab*-paclitaxel in both the metastatic and early-stage settings.

## Review

### Methodology

A literature search for clinical trial publications and presentations from 2004 through 2014 of data on *nab*-paclitaxel used to treat BC in the neoadjuvant, adjuvant, or metastatic setting was carried out using PubMed and abstract search engines from the American Society of Clinical Oncology and the San Antonio Breast Cancer Symposium. Search terms included ‘*nab*-paclitaxel’, ‘albumin-bound paclitaxel’, ‘abi-007’, ‘Abraxane’, and ‘breast cancer’. Reports were selected if they were from phase II or III trials and described *nab*-paclitaxel monotherapy or *nab*-paclitaxel in combination with targeted agents. Combinations that included other cytotoxic agents were excluded.

### Results

#### *nab*-Paclitaxel monotherapy in metastatic breast cancer

The literature search revealed five trials of *nab*-paclitaxel monotherapy in MBC that fit the criteria described above (phase II, *n* = 4; phase III, *n* = 1; Table 1).

**Table 1:** *nab*-Paclitaxel monotherapy in metastatic breast cancer

Trial	Phase	Patient population	*nab*-Paclitaxel regimen	Patients receiving protocol-specified dose (%)	Efficacy outcomes			Select grade 3/4 adverse events (%)		
					ORR (%)	PFS (months)	OS (months)	Neutropenia	Neuropathy	Fatigue
Ibrahim and colleagues, 2005 [19]	II	CN (*n* = 15), PT (*n* = 48)	300 mg/m^2^ q3w	75^a^	48 (CN 64; PT 21)	TTP 6.1	14.6	51	11^b^	13^b^
Gradishar and colleagues, 2005 [20]	III	CN (*n* = 97), PT (*n* = 132)	260 mg/m^2^ q3w	96 (received 90 % of protocol-specified dose)	33 (CN 42; PT 27)	TTP 5.3	15.0	30^c^	10^b^	<10^b,c^
Guan and colleagues, 2009 [27]	II	Chinese; CN (*n* = 61), PT (*n* = 43)	260 mg/m^2^ q3w	≥95	54 (CN 56; PT 51)	7.6	17.8	42	7^b^	NR
Blum and colleagues, 2007 [28]	II	PT	100 mg/m^2^ qw 3/4 (*n* = 106)	87	14	3.0	9.2	17	8^b^	5^b^
			125 mg/m^2^ qw 3/4 (*n* = 75)	68	16	3.5	9.1	32	19^b^	12^b^
Gradishar and colleagues, 2009 and 2012 [29, 30]	II	CN	300 mg/m^2^ q3w (*n* = 76)	80	37	11.0	27.7	43	21^b^	5^b^
			100 mg/m^2^ qw 3/4 (*n* = 76)	82	45	12.8	22.2	25	9^b^	0
			150 mg/m^2^ qw 3/4 (*n* = 74)	53	49	12.9	33.8	45	22^b^	4^b^

^a^Percentage of patients without *nab*-paclitaxel dose reductions. ^b^No grade 4 event. ^c^Estimated from a bar graph in the publication. CN, chemotherapy-naïve; NR, not reported; ORR, overall response rate; OS, overall survival; PFS, progression-free survival; PT, previously treated; q3w, every 3 weeks; qw 3/4, during the first 3 of 4 weeks; TTP, time to progression

Ibrahim and colleagues evaluated *nab*-paclitaxel 300 mg/m^2^ q3w in a phase II trial of women with MBC (*n* = 15 chemotherapy naïve and *n* = 48 previously treated) [19]. The ORR was 48 %. Dose reductions to 225 mg/m^2^ occurred in 16 patients (25 %) due to neutropenia (*n* = 7), sensory neuropathy (*n* = 4), febrile neutropenia (*n* = 3), myalgia (*n* = 3), and fatigue (*n* = 2). Seven patients (11 %) discontinued treatment, primarily resulting from sensory neuropathy, which occurred in five patients. *nab*-Paclitaxel 300 mg/m^2^ q3w demonstrated substantial antitumor activity with a manageable safety profile in this patient population.

A pivotal 2005 phase III trial demonstrated the superiority of *nab*-paclitaxel 260 mg/m^2^ q3w versus sb-paclitaxel 175 mg/m^2^ q3w in a patient population in which the majority had received zero or one prior line of therapy for MBC [20]. The ORR for all patients was significantly higher with *nab*-paclitaxel treatment versus sb-paclitaxel (33 % vs 19 %, *P* = 0.001). In the *nab*-paclitaxel treatment group, the ORR was similar in groups defined by age (34 % in patients aged <65 years and 27 % in patients aged ≥65 years) or by the presence or absence of visceral dominant disease (both 34 %). Tumor responses were also observed in chemotherapy-naïve and previously treated patients (42 % and 27 %, respectively) and patients with prior anthracycline exposure in either the adjuvant or metastatic setting (34 %). The incidence of grade 4 neutropenia was low with *nab*-paclitaxel treatment, and although grade 3 sensory neuropathy occurred in 10 % of patients, improvements to grade 2 or lower occurred in a median of 22 days. There were no safety differences in younger versus older patients treated with *nab*-paclitaxel.

Additional evidence for the safety and efficacy of *nab*-paclitaxel monotherapy for MBC was provided by a phase II trial in Chinese patients [27]. Like participants in the pivotal 2005 phase III trial described above [20], chemotherapy-naïve and previously treated patients with MBC received the US Food and Drug Administration-recommended starting dose of *nab*-paclitaxel (260 mg/m^2^ q3w) or sb-paclitaxel 175 mg/m^2^ q3w. Patients in each treatment arm received ≥95 % of the protocol dose. The ORR was significantly higher with *nab*-paclitaxel than with sb-paclitaxel in the overall population (54 % vs 29 %, *P* < 0.001), as well as in young patients, chemotherapy-naïve patients, patients with no prior anthracycline therapy, patients with ≤3 or >3 metastatic lesions, and patients with visceral disease.

A phase II trial in patients with heavily pretreated MBC examined the efficacy of *nab*-paclitaxel at 100 mg/m^2^ (*n* = 106) and 125 mg/m^2^ (*n* = 75), both qw 3/4 [28]. Patients had received a median of three previous chemotherapy regimens for metastatic disease. Efficacy results were similar between the treatment groups, with ORRs of 14 % and 16 % for the 100 mg/m^2^ and 125 mg/m^2^ treatment arms, respectively. Median progression-free survival (PFS) was 3 months for the lower dose of *nab*-paclitaxel and 3.5 months for the higher dose. Median OS was also similar between cohorts (9.2 and 9.1 months, respectively).

In a more recent, randomized phase II trial, *nab*-paclitaxel monotherapy was administered at 300 mg/m^2^ q3w, 100 mg/m^2^ qw 3/4, or 150 mg/m^2^ qw 3/4 as first-line treatment for patients with MBC [29, 30]. A docetaxel arm was also included. Patients treated with the higher dose of *nab*-paclitaxel qw 3/4 had a significantly longer OS than those treated with the lower dose on the same schedule (*P* = 0.008). Median OS was 33.8 months in patients who received *nab*-paclitaxel 150 mg/m^2^ qw 3/4, compared with 22.2 and 27.7 months in patients who received 100 mg/m^2^ qw 3/4 and 300 mg/m^2^ q3w, respectively. The ORRs (primary endpoint) for *nab*-paclitaxel 300 mg/m^2^ q3w, 100 mg/m^2^ qw 3/4, and 150 mg/m^2^ qw 3/4 were 37 %, 45 %, and 49 %, respectively, by independent assessment and were 46 %, 63 %, and 74 % by investigator assessment (35 % and 39 %, respectively, for docetaxel). Grade 3/4 neutropenia and grade 3 sensory neuropathy were significantly more frequent in patients treated with *nab*-paclitaxel doses of 300 mg/m^2^ q3w and 150 mg/m^2^ qw 3/4 than 100 mg/m^2^ qw 3/4. Dose reductions (by 20 %, per protocol) were more frequent in the 150 mg/m^2^ qw 3/4 cohort (47 %) than in the other treatment arms (18 % and 20 % in the 100 mg/m^2^ qw 3/4 and the 300 mg/m^2^ q3w arms, respectively). More dose delays also occurred in the 150 mg/m^2^ qw 3/4 arm (81 %) than in the 100 mg/m^2^ qw 3/4 and 300 mg/m^2^ q3w arms (45 % and 43 %, respectively). Nonetheless, the median treatment duration was longest in patients treated with 150 mg/m^2^*nab*-paclitaxel qw 3/4 (38 weeks vs 22 weeks in the 300 mg/m^2^ q3w arm (*P* = 0.001) and 30 weeks in the 100 mg/m^2^ qw 3/4 arm (*P* = not significant)). Thus, it appears that careful dose modification was a successful strategy to allow further treatment in the 150 mg/m^2^ arm of this phase II trial. In the 150 mg/m^2^ qw 3/4 treatment arm, best responses occurred at cycle 2 and dose reductions occurred at a median of cycle 4, indicating that patients may be able to experience a response with this regimen before experiencing significant toxicity that requires a dose reduction. Therefore, 150 mg/m^2^ qw 3/4 may be an appropriate dose of *nab*-paclitaxel for patients who need a rapid tumor response. However, the toxicity observed may make such a dose less desirable to other patients.

#### *nab*-Paclitaxel in combination with targeted agents in metastatic breast cancer

According to search criteria, five trials have evaluated combination therapy with *nab*-paclitaxel and targeted agents for patients with MBC (phase II, *n* = 4, phase III, *n* = 1; Table 2).

**Table 2:** ***nab*** **-Paclitaxel in combination with select targeted therapies in metastatic breast cancer**

Trial	Phase	Patient population	*nab*-Paclitaxel regimen	Patients receiving protocol-specified dose (%)	Efficacy outcomes			Select grade 3/4 adverse events (%)		
					ORR (%)	PFS (months)	OS (months)	Neutropenia	Neuropathy	Fatigue
**Bevacizumab combinations**										
Seidman and colleagues, 2013 [31]	II	CN	130 mg/m^2^ qw + bev 10 mg/kg q2w (*n* = 79)	33^a^	46	8.8	23.7	33	46	19
			260 mg/m^2^ q2w + bev 10 mg/kg q2w (*n* = 54)^b^	39^a^	41	5.8	19.0	6	56	35
			260 mg/m^2^ q3w + bev 15 mg/kg q3w (*n* = 75)	53	45	7.7	21.3	16	33	17
Danso and colleagues, 2008 [32]	II	CN	125 mg/m^2^ qw 3/4 + bev 10 mg/kg q2w (*n* = 50)	NR	33	7.4	NR	50	13^c^	13^c^
Rugo and colleagues, 2012 [33]	III	CN	150 mg/m^2^ qw 3/4 + bev 10 mg/kg q2w (*n* = 271)	NR^d^	NR	9.2	27	47	25	16
**HER2-targeted therapy combinations**										
Mirtsching and colleagues, 2011 [34]	II	CN	125 mg/m^2^ qw 3/4 (HER2-/unknown; *n* = 50), 125 mg/m^2^ qw 3/4 + trastuz (HER2+; *n* = 22)	100^e^	42 (HER2-negative 38.1; HER2-positive 52.4)	14.5 (HER2-negative 12.8; HER2-positive 18.7)	29 (HER2-negative 27.3; HER2-positive 36.8)	8^f^	6^f^	5^f^
Yardley and colleagues, 2013 [35]	II	CN and PT (second line)	100 mg/m^2^ qw 3/4 + lapatinib (*n* = 60)^g^	78^a^	53	9.1	Not reached	22^f^	3^f^	10

^a^Percentage of patients without *nab*-paclitaxel dose reductions. ^b^Arm closed early due to toxicity. ^c^Estimated from a bar graph in the publication. ^d^Forty-five percent of patients had a dose reduction by cycle 3. ^e^Median dose intensity was 100 % of planned dose. Dose modifications due to toxicity were required in 19 % of patients; the type of modification was not specified. ^f^No grade 4 event. ^g^Original dose was modified after toxicity in first few patients. bev, bevacizumab; CN, chemotherapy-naïve; HER2, human epidermal growth factor receptor 2; NR, not reported; ORR, overall response rate; OS, overall survival; PFS, progression-free survival; PT, previously treated; q2w, every 2 weeks; q3w, every 3 weeks; qw, weekly; qw 3/4, during the first 3 of 4 weeks; trastuz, trastuzumab

##### Bevacizumab

A recent phase II trial tested three schedules of *nab*-paclitaxel treatment in combination with bevacizumab as first-line treatment for patients with MBC: *nab*-paclitaxel 130 mg/m^2^ weekly (uninterrupted weekly), 260 mg/m^2^ every 2 weeks (q2w; dose-dense), and 260 mg/m^2^ q3w [31]. The primary endpoint of the ORR was similar across treatment groups (46 %, 41 %, and 45 % for *nab*-paclitaxel 130 mg/m^2^ weekly, *nab*-paclitaxel 260 mg/m^2^ q2w, and *nab*-paclitaxel 260 mg/m^2^ q3w, respectively), and nonsignificant trends in PFS and OS (secondary endpoints) favored weekly dosing. The dose-dense *nab*-paclitaxel 260 mg/m^2^ arm was closed early due to toxicity, and therefore this regimen was not deemed viable. There were more dose reductions and delays with uninterrupted weekly *nab*-paclitaxel treatment than with the 260 mg/m^2^ q2w or q3w treatments (reductions 67 % vs 61 % vs 47 % and delays 86 % vs 52 % vs 53 %, respectively), primarily resulting from peripheral neuropathy. Although there were no significant differences in response rates, nonsignificant trends in secondary endpoints from this study seemed to favor uninterrupted weekly *nab*-paclitaxel treatment rather than the other regimens tested. However, due to the high rate of dose reductions and delays seen in this trial and given findings from other trials, a schedule qw 3/4 may be preferable [28, 29].

Two other trials have explored a *nab*-paclitaxel regimen qw 3/4 in combination with bevacizumab for treatment of MBC. A phase II study of *nab*-paclitaxel 125 mg/m^2^ qw 3/4 plus bevacizumab demonstrated the feasibility of this schedule, although the incidence of grade 3/4 neutropenia was 50 % (Table 2; grade 4, 16 %) [32]. A higher dose of *nab*-paclitaxel qw 3/4 (150 mg/m^2^) was compared with sb-paclitaxel qw 3/4, both in combination with bevacizumab, in the phase III Cancer and Leukemia Group B 40502 trial that was reported at the 2012 annual meeting of the American Society for Clinical Oncology [33]. A third arm of weekly ixabepilone plus bevacizumab was also included, but the sb-paclitaxel plus bevacizumab arm was designated the control arm. Neither PFS (median 9.2 vs 10.6 months for *nab*-paclitaxel plus bevacizumab vs sb-paclitaxel plus bevacizumab, respectively, *P* = 0.12) nor OS (27 vs 26 months, *P* = 0.92) were different between the groups. Treatment with *nab*-paclitaxel 150 mg/m^2^ plus bevacizumab was associated with higher rates of grade 3/4 hematologic (51 % vs 21 %, *P* < 0.0001) and nonhematologic (60 % vs 44 %, *P* = 0.0002) toxicities compared with sb-paclitaxel plus bevacizumab. These higher rates of toxicities and a higher rate of dose reductions (45 % vs 15 % at cycle 3) and dose discontinuations (approximately 22 % vs 15 % at cycle 3 and 48 % vs 15 % at cycle 5) suggest that this *nab*-paclitaxel dose may be too high for combination with bevacizumab in unselected patients with MBC.

##### Human epidermal growth factor receptor 2-targeted therapy

In a phase II trial, *nab*-paclitaxel 125 mg/m^2^ qw 3/4 with or without trastuzumab demonstrated efficacy and a favorable safety profile for first-line treatment of patients with MBC [34]. Patients whose tumors overexpressed human epidermal growth factor receptor 2 (HER2-positive tumors) received *nab*-paclitaxel plus trastuzumab; patients with HER2-negative tumors received *nab*-paclitaxel only. Treatment resulted in a 42.2 % ORR (52.4 % for patients with HER2-positive tumors and 38.1 % for patients with HER2-negative tumors), and the PFS and OS for the intent-to-treat population were 14.5 months and 29 months, respectively. When necessary, the *nab*-paclitaxel dose was reduced (to 100 mg/m^2^ and then to 80 mg/m^2^) as a result of toxic effects, and dose modifications due to toxicity occurred in 19.4 % of patients. This protocol resulted in a favorable safety profile: the most common grade 3 adverse events were pain (11 %), infection (9 %), neutropenia (8 %), sensory neuropathy (6 %), and fatigue (5 %). Only one grade 4 cardiac event was reported.

A single-arm phase II study also evaluated the same *nab*-paclitaxel dose and schedule (weekly *nab*-paclitaxel 125 mg/m^2^ qw 3/4) combined with lapatinib 1,250 mg daily for treatment of patients with HER2-positive MBC [35]. A safety analysis in the first five patients enrolled found grade 3 toxicities in all patients after one cycle of treatment, with four patients having experienced neutropenia and one patient having experienced neutropenic fever and diarrhea. Doses of both study drugs were subsequently reduced, with remaining patients given *nab*-paclitaxel 100 mg/m^2^ and lapatinib 1,000 mg instead. Treatment resulted in an ORR of 53 %, with the majority of patients demonstrating a partial response (47 %). *nab*-Paclitaxel dose delays occurred in 25 % of patients, and 55 % of patients missed *nab*-paclitaxel doses, both primarily due to nonhematologic toxicity. The most common grade 3/4 adverse events were diarrhea (22 %) and neutropenia (22 %). A regimen of weekly *nab*-paclitaxel 100 mg/m^2^ qw 3/4 in combination with lapatinib is thus a feasible treatment in this patient population.

#### *nab*-Paclitaxel in early breast cancer

Various doses and schedules of *nab*-paclitaxel have also been evaluated in patients with early-stage BC (phase III, *n* = 1; phase II, *n* = 6; Table 3).

**Table 3:** ***nab*** **-Paclitaxel in patients with early-stage breast cancer**

Trial	Phase	Patient population	*nab*-Paclitaxel regimen	Patients receiving protocol-specified dose (%)	Efficacy outcomes		Select grade 3/4 adverse events (%)		
					pCR in breast and LNs (%)	Other parameters	Neutropenia	Neuropathy	Fatigue
**Neoadjuvant**									
Untch and colleagues (GeparSepto), 2014 [36]	III	Unselected (*n* = 1,204)	*nab-*P 125 mg/m^2^ qw × twelve cycles → EC q3w × four cycles (+ trastuz and pertuz throughout for HER2+)	NR	38	NR	61	10	6
Nahleh and colleagues (S0800), 2014 [37]	II	HER2– IBC or LABC (*n* = 200)	Bev + *nab*-P 100 mg/m^2^ qw × 12 → AC + peg q2w × six cycles	NR	36	NR	NR	NR	NR
			*nab*-P 100 mg/m^2^ qw × twelve cycles followed or preceded by AC + peg q2w × six cycles		21				
Martin and colleagues (GEICAM), 2013/2014 [38, 39]	II	HR+, HER2− (*n* = 81)	*nab*-P 150 mg/m^2^ qw 3/4 (monotherapy)	70	7.4	RCB 0 + 1 = 25 %; ORR = 77 %	16	3^a^	4^a^
Robidoux and colleagues, 2010 [41]	II	Unselected (*n* = 66)	*nab*-P 100 mg/m^2^ qw × twelve cycles → FEC q3w × four cycles (+ trastuz for HER2+)	98	26	cCR = 12 %; estimated 2-year PFS = 81 %	3^a^	5^a^	6^a^
Zelnak and colleagues, 2012 [43]	II	HER2+ (*n* = 27)	*nab*-P 260 mg/m^2^ q2w × four cycles → vin + trastuz	NR	48	ORR = 100 %; cCR = 74 %	6^a,b^	3^a,b^	1^a,b^
**Adjuvant**									
McArthur and colleagues, 2011 [44]	II	HER2− (*n* = 80)	AC + peg + bev → *nab*-P 260 mg/m^2^ q2w + bev × four cycles → bev	NR	NR	4^a^	14^a^	10^a^	
Pippen and colleagues, 2011 [45]	II	HER2− (*n* = 197)	AC + peg + bev → *nab*-P 260 mg/m^2^ q2w + bev × four cycles → bev	91	NR	8	5	13	

^a^No grade 4 event. ^b^Estimated from a bar graph in the publication. AC, doxorubicin/cyclophosphamide; bev, bevacizumab; cCR, clinical complete response; EC, epirubicin/cyclophosphamide; FEC, 5-fluorouracil/epirubicin/cyclophosphamide; HER2–, human epidermal growth factor receptor 2-negative; HER2+, human epidermal growth factor receptor 2-positive; HR+, hormone receptor-positive; IBC, inflammatory breast cancer; LABC, locally advanced breast cancer; LN, lymph node; *nab*-P, *nab*-paclitaxel; NR, not reported; ORR, overall response rate; pCR, pathologic complete response; peg, pegfilgrastim; pertuz, pertuzumab; PFS, progression-free survival; q2w, once every 2 weeks; q3w, once every 3 weeks; qw, once weekly; qw 3/4, during the first 3 of 4 weeks; RCB, residual cancer burden; trastuz, trastuzumab; vin, vinorelbine

In the neoadjuvant setting, the treatment goal is rapid reduction of tumor size in order to facilitate surgical removal and achieve successful breast conservation. The recent large phase III GeparSepto trial evaluated *nab*-paclitaxel as neoadjuvant treatment for patients with early BC [36]. Patients received *nab*-paclitaxel 125 mg/m^2^ continuous weekly (reduced from the initial dose of 150 mg/m^2^ after a protocol amendment due to neurotoxicity; M Untch, oral communication, February 2015) or sb-paclitaxel 80 mg/m^2^ continuous weekly, each followed by epirubicin/cyclophosphamide. Patients with HER2-positive tumors also received trastuzumab and pertuzumab throughout treatment. The pathological complete response (pCR) rate was significantly higher with *nab*-paclitaxel compared with sb-paclitaxel (38 % vs 29 %, *P* = 0.001), and this effect was seen in all subgroups, including patients with triple-negative tumors (that is, hormone receptor-negative and HER2-negative; *n* = 275, 48.2 % vs 25.7 %, *P* < 0.001). *nab*-Paclitaxel was associated with significantly more grade 3/4 peripheral sensory neuropathy compared with sb-paclitaxel (10 % vs 3 %, *P* < 0.001). Long-term follow-up would be necessary to confirm whether the higher pCR translates into improved survival.

Also in the neoadjuvant setting, the S0800 phase II safety and efficacy trial evaluated bevacizumab with *nab*-paclitaxel 100 mg/m^2^ weekly followed by dose-dense AC with pegfilgrastim, compared with nonbevacizumab arms containing *nab*-paclitaxel followed or preceded by dose-dense AC with pegfilgrastim, in patients with HER2-negative inflammatory or locally advanced BC [37]. The addition of bevacizumab resulted in a significantly higher pCR rate (primary endpoint 36 % vs 21 % in the nonbevacizumab arms, exact *P* = 0.021), especially for patients with triple-negative tumors (59 % vs 28 %, *P* = 0.014). The 3-year OS for the bevacizumab arm versus the nonbevacizumab arm was 87 % versus 83 % (*P* = 0.59). Grade 3/4 events were common and similar between groups (67 % and 65 % in the bevacizumab and nonbevacizumab arms, respectively).

Neoadjuvant treatment with four cycles of single-agent *nab*-paclitaxel 150 mg/m^2^ qw 3/4 was safe and effective in the phase II GEICAM trial in patients with early-stage, hormone receptor-positive, HER2-negative BC [38, 39]. Treatment resulted in a clinical response rate of 77 %. Dose reductions occurred in 30 % of patients, 12 % for neutropenia and 16 % for neuropathy. The most common grade 3/4 toxicity was neutropenia (16 %), and 25 % of patients had no residual cancer cells or only microscopic residual disease burden (residual cancer burden 0 or 1 by Symann’s classification [40]). Forty percent of patients underwent breast-conserving surgery.

A phase II trial demonstrated that weekly *nab*-paclitaxel 100 mg/m^2^ for 12 weeks followed by 5-fluorouracil/epirubicin/cyclophosphamide is a safe and effective neoadjuvant treatment option for patients with locally advanced breast cancer [41]. Patients with HER2-positive tumors received concomitant trastuzumab. Only 2 % of the planned *nab*-paclitaxel doses were reduced or skipped. A pCR was observed in 58 % of patients with HER2-positive tumors, in 11 % of patients with hormone receptor-positive, HER2-negative tumors, and in 28 % of patients with triple-negative tumors. Breast-conserving surgery was performed in 58 % of patients, and *nab*-paclitaxel treatment resulted in low rates of grade 3 toxicities and no grade 4 events. A similar regimen is currently being explored in a phase III trial of high-risk patients with HER2-negative tumors [42].

Sequential neoadjuvant treatment with dose-dense *nab*-paclitaxel followed by a cytotoxic and targeted agent combination had significant activity and a manageable safety profile in patients with HER2-positive early BC [43]. In this phase II trial, patients (*n* = 27) received *nab*-paclitaxel 260 mg/m^2^ q2w for four cycles followed by vinorelbine and trastuzumab. This treatment resulted in a high rate of pCR (48 %) and was well tolerated, with low rates of grade 3 events and no grade 4 events. The main toxicities observed were neuropathy, myalgia/arthralgia, and fatigue, but events were primarily grade 1 or grade 2.

In the adjuvant setting, two phase II studies have demonstrated the feasibility and safety of dose-dense *nab*-paclitaxel regimens in early BC (Table 3). A phase II trial (*n* = 80) evaluated sequential treatment with dose-dense AC plus bevacizumab, followed by four cycles of dose-dense *nab*-paclitaxel 260 mg/m^2^ plus bevacizumab, and, finally, treatment with bevacizumab alone [44]. This study met its primary endpoint of cardiac safety, as the rate of cardiac events was low. Another phase II trial evaluated dose-dense AC plus pegfilgrastim for four cycles, followed by either *nab*-paclitaxel 260 mg/m^2^ q2w or sb-paclitaxel 175 mg/m^2^ q2w (cycles five to eight) [45]. Dose-dense bevacizumab was administered with AC, *nab*-paclitaxel, or sb-paclitaxel, and then alone for 10 more cycles q3w. Despite a 44 % higher cumulative paclitaxel dose for *nab*-paclitaxel compared with sb-paclitaxel (*P* < 0.0001), the safety profile between the two regimens was similar except for significantly more grade 3/4 leukopenia in the sb-paclitaxel arm. Phase III trials are necessary to confirm these feasibility and tolerability findings and to evaluate efficacy in adjuvant BC.

## Conclusions

*nab*-Paclitaxel is approved for MBC at a dose of 260 mg/m^2^ q3w [2]. However, numerous studies have suggested that a schedule qw 3/4 could also be a reasonable option. In fact, a phase II trial demonstrated better ORRs for qw 3/4 regimens (100 mg/m^2^ and 150 mg/m^2^) versus a q3w regimen as monotherapy [29, 30]. The 100 mg/m^2^ dose demonstrated a more manageable tolerability profile compared with either of the other regimens, with lower rates of grade 3/4 neutropenia, sensory neuropathy, and fatigue, whereas the 150 mg/m^2^ dose demonstrated the longest OS, albeit with considerably more toxicity [30].

Some data in this review call into question whether intense regimens of *nab*-paclitaxel are the most appropriate approach for MBC, particularly in combination with bevacizumab, since delaying disease progression is the main aim of therapy. High rates of dose modification and discontinuation were observed with *nab*-paclitaxel 150 mg/m^2^ monotherapy qw 3/4 in a phase II trial and with *nab*-paclitaxel 150 mg/m^2^ qw 3/4 plus bevacizumab in a phase III trial [29, 30, 33]. Toxicity also limited delivery of *nab*-paclitaxel at 260 mg/m^2^ q2w and 130 mg/m^2^ weekly uninterrupted, both in combination with bevacizumab, in a phase II trial [31].

In contrast to treatment for MBC, *nab*-paclitaxel at 260 mg/m^2^ q2w plus bevacizumab for early-stage disease was feasible [31, 44, 45]. The question of whether a drug-drug interaction exists between *nab*-paclitaxel and bevacizumab has not been examined. However, it is unlikely that future MBC trials will test the combination since the US Food and Drug Administration approval of bevacizumab for MBC has been withdrawn [46].

Patients who were treated with neoadjuvant *nab*-paclitaxel 150 mg/m^2^ qw 3/4 in the GEICAM 2011–2012 trial experienced less toxicity compared with the patients treated with the same regimen in the metastatic setting described above. The only grade 3/4 toxicity reported by >5 % of patients in the neoadjuvant trial was neutropenia (16 %), and the median relative dose intensity was 98.5 % [39]. Grade 3 sensory neuropathy occurred in two patients (2.5 %), and there were no cases of grade 4 neuropathy [39]. Furthermore, the regimen was effective, eliciting a clinical response rate of 77 % and a 25 % rate of residual cancer burden 0 or 1 [38]. In the GeparSepto trial, the weekly *nab*-paclitaxel dose had to be reduced from 150 mg/m^2^ to 125 mg/m^2^, resulting in a 38 % pCR rate and grade 3/4 sensory neuropathy in 10 % of patients [36]. Thus, although the 150 mg/m^2^ qw 3/4 dose may be questionable in MBC, results were mixed for this regimen as a neoadjuvant treatment option.

Numerous studies have revealed substantial clinical activity for *nab*-paclitaxel in the metastatic setting, and a growing number of reports suggest similar activity in early-stage disease. Optimizing regimens in BC treatment depends on a number of patient-specific and disease-specific factors. Although multiple schedules and doses have demonstrated feasibility and activity, single-agent *nab*-paclitaxel may be preferable to combination therapy for unselected patient populations. Ongoing and future trials will reveal whether combination therapies are advantageous for patients with aggressive disease subtypes [47].

## Abbreviations

AC: Doxorubicin/cyclophosphamide
BC: Breast cancer
HER2: Human epidermal growth factor receptor 2
MBC: Metastatic breast cancer
ORR: Overall response rate
OS: Overall survival
pCR: Pathologic complete response
PFS: Progression-free survival
q2w: Every 2 weeks
q3w: Every 3 weeks
qw 3/4: During the first 3 of 4 weeks
sb-paclitaxel: Solvent-based paclitaxel
